# Clostridium difficile-associated diarrhea in radiooncology: an underestimated problem for the feasibility of the radiooncological treatment?

**DOI:** 10.1186/1748-717X-6-89

**Published:** 2011-08-01

**Authors:** Matthias G Hautmann, Matthias Hipp, Oliver Kölbl

**Affiliations:** 1 Institutional address: Department of Radiotherapy, University of Regensburg, Regensburg, Germany

**Keywords:** Clostridium difficile-associated diarrhea, Clostridium difficile, Diarrhea, Colitis, Radiotherapy, Radiation Therapy, Chemoradiation

## Abstract

**Background and Purpose**

Over the last years an increasing incidence of Clostridium difficile-associated diarrhea (CDAD) has been reported. Especially haematology-oncology patients are at risk of developing CDAD.

The aim of this analysis is to determine the incidence of CDAD in radiooncological patients and to find out what relevance CDAD has for the feasibility of the radiooncological treatment, as well as to detect and describe risk factors.

**Patients and Methods**

In a retrospective analysis from 2006 to 2010 34 hospitalized radiooncological patients could be identified having CDAD. The risk factors of these patients were registered, the incidence was calculated and the influence on the feasibility of the radiooncological therapy was evaluated. Induced arrangements for prophylaxis of CDAD were identified and have been correlated with the incidence.

**Results**

The incidence of CDAD in our collective is 1,6%. Most of the patients suffering from a CDAD were treated for carcinoma in the head and neck area. Common risk factors were antibiotics, proton pump inhibitors, cytostatic agents and tube feeding.

Beside a high rate of electrolyte imbalance and hypoproteinemia a decrease of general condition was frequent. 12/34 patients had a prolonged hospitalization, in 14/34 patients radiotherapy had to be interrupted due to CDAD. In 21 of 34 patients a concomitant chemotherapy was planned. 4/21 patients could receive all of the planned cycles and only 2/21 patients could receive all of the planned cycles in time.

4/34 patients died due to CDAD. In 4/34 patients an initially curative treatment concept has to be changed to a palliative concept.

With intensified arrangements for prophylaxis the incidence of CDAD decreased from 4,0% in 2007 to 0,4% in 2010.

**Conclusion**

The effect of CDAD on the feasibility of the radiotherapy and a concomitant chemotherapy is remarkable. The morbidity of patients is severe with a high lethality.

Reducing of risk factors, an intense screening and the use of probiotics as prophylaxis can reduce the incidence of CDAD.

## Background and Purpose

Clostridium difficile (CD) appears normally as a harmless environmental gram positive anaerobic bacteria which becomes pathogen in several circumstances [1,2]. Clostridium difficile can be isolated from the stool of up to five per cent of healthy adults. Some strains produce toxin and can therefore cause diarrhea [3]. CD is the aetiological agent for most of the cases of pseudo membranous colitis. Over the last years an increasing incidence of Clostridium difficile-associated diarrhea (CDAD) has been reported. Furthermore, more severe courses of the disease have been described because of new virulent strains [3-6].

Several risk factors for CDAD are known. Beside antibiotic intake, other drugs like immunosuppressant, cytostatic agents and proton pump inhibitors (PPI) have been identified to trigger CDAD [5,7-10]. Also tube feeding, parenteral nutrition as well as a reduced general condition and compromised immune function have been described as risk factors [1,2,11].

Especially haematology-oncology patients are at risk of developing CDAD [12-15]. Those haematology-oncology patients often have systemic diseases and in many cases receive high dosed chemotherapy.

Radiooncological patients are mostly suffering from localised tumour and receive radiotherapy alone or with a moderate dosed concomitant chemotherapy compared to chemotherapy of haematology patients. Because of the mainly local therapy radiooncological patients have higher local toxicity. Especially stomatitis, mucositis and dysphagia are common in radiooncological patients and might be relevant as risk factors.

In summary a lot of radiooncological patients have several risk factors. Beside concomitant chemotherapy, the frequency of a treatment with PPI and antibiotics is estimated to be high on a radiooncological ward [16-19]. Also tube feeding and parenteral nutrition is common [20-22].

CDAD has a lethality of 0.5% to 2.0% and an increasing morbidity [3,14]. A high morbidity and a negative influence on the treatment of the underlying disease have been documented, especially for surgical patients or patients on intensive care units [23,24]. A high number of acute renal failure, weight loss, electrolyte imbalance and hypoproteinemia have been described [5,23].

The influence of CDAD for the treatment of oncological patients is not well reviewed. Because of the existing data, multiple problems for the treatment of those patients can be assumed [25,26].

Often inpatient stay is prolonged because of CDAD. The costs for the health care system are high. There are data showing estimated additional costs between 5243 US$ and 8570 US$ in Europe per patient with a primary episode of CDAD and over 13600 US$ for a case of recurrent CDAD [5,27].

Referring to this data, there might be a negative influence on the feasibility of a radiooncological treatment for patients suffering from a CDAD.

The aim of this analysis is to determine the incidence of CDAD in radiooncological patients and to find out what relevance CDAD has for the feasibility of the radiooncological treatment, as well as to detect and describe risk factors.

## Patients and Methods

The study was performed for patients of a department of radiotherapy of a German university hospital. In- and outpatients were looked up for CDAD. Only inpatients could be identified developing CDAD during radiooncological treatment.

In a retrospective analysis from 2006 to 2010 34 hospitalized radiooncological patients could be identified having CDAD. In that time 2150 patients were on the radiooncological ward in total (484 in 2006, 398 in 2007, 423 in 2008, 384 in 2009 and 461 in 2010).

Radiooncological inpatients were identified from CD positive patients registered in the department for microbiology and hygiene. Beside that, the enquiry was done by checking codification for final payment. All patients had to have at least one stool sample being tested positive for CD toxin A or B.

Until the year 2008 all inpatients with watery diarrhea of more than 24 hours and all patients with medical history of antibiotic intake and non-watery diarrhea of more than 48 hours were screened for CDAD. Beside that all patients with any diarrhea of more than 72 hours were screened for CDAD. For all patients a stool sample was collected and screened for Clostridium difficile toxin A and B by enzyme immunoassay.

Since 2008 for all patients with any diarrhea longer than 24 hours were screened for CD toxin A and B. In case of clinical suspicion of CDAD and negative findings for CD toxin A or B, two more stool samples were screened by enzyme immunoassay.

Since 2009 for all patients a stool sample was cultured for toxigenic CD additionally to the enzyme immunoassay.

All patients with positive findings for CD toxin A or B had clinical symptoms of CDAD. In our collective no asymptomatic carrier could be identified.

In case of suspicion of a relapse of CDAD, beside screening for CD toxin A and B, a sigmoidoscopy was performed and a stool sample was cultured for toxigenic CD.

The risk factors of the patients were registered. Antibiotics and cytostatic agents applicated up to four weeks prior to diagnosis of CDAD were assessed. Patients with a long time medication of immunosuppressive drugs or patients with a Leukopenia CTC grade IV were counted as immunosupressed. The influence on the feasibility of the radiooncological therapy in terms of interruption or abandonment of the radiotherapy, change of therapy concept and the effect on the feasibility of chemotherapy were evaluated.

Induced arrangements for prophylaxis of CDAD were identified and have been correlated with the incidence.

## Results

### Patient's characteristics

The incidence of CDAD in our collective is 1,6% (34 of 2150 patients). Of the 34 patients having a CDAD, 24 were male, 10 were female. The median age was 67 Years with a standard deviation of 10 Years (Range from 46 to 85 years).

Most patients suffered from a head and neck cancer or metastases in the head and neck area.

Treatment areas were in 21 cases the head and neck. 7 patients were treated thoracically, 5 pelvic and one abdominal (Table 1).

**Table 1:** Patient characteristics

	sex	Underlying disease
1	F	Anaplastic thyroid carcinoma
2	M	Squamous cell carcinoma of the head and neck (SCCHN)
3	F	Thyroid metastasis of an renal cell carcinoma
4	M	SCCHN
5	F	Cervical carcinoma
6	M	SCCHN
7	M	Bronchial carcinoma with a simultaneous SCCHN
8	F	SCCHN
9	M	Bronchial carcinoma
10	M	SCCHN
11	M	SCCHN
12	F	SCCHN
13	M	Bronchial carcinoma
14	M	M. Hodgkin
15	M	Bronchial carcinoma
16	M	SCCHN
17	M	Anal carcinoma
18	M	Bronchial carcinoma
19	F	Giant cell B-NHL with an abdominal bulk
20	M	SCCHN
21	F	Rectal carcinoma
22	M	SCCHN
23	M	SCCHN
24	M	SCCHN
25	M	Oesophageal carcinoma
26	M	SCCHN
27	F	SCCHN
28	M	SCCHN
29	F	Cervical carcinoma
30	M	Pelvine bone metastasis of a rectal carcinoma
31	M	Oesophageal carcinoma
32	F	SCCHN
33	M	SCCHN
34	M	SCCHN

The average treatment time was 41 days with a standard deviation of 19 days (Range from 5 to 74 days). The average time as inpatients was 36 days with a standard deviation of 22 days (Range from 2 to 80 days).

### Risk factors

The following data in terms of risk factors were collected: The median pre treatment Karnofsky Performance Status (KPS) of the patients was 70% (range from 40% to 100%).

Twenty patients out of 34 (59%) had an antibiotic treatment up to four weeks before developing diarrhea.

The most common antibiotic being taken was Piperacillin/Combactam with 29% of all patients (10/34 patients) and 50% of the patients having an antibiotic treatment (10/20). Beside Piperacillin/Combactam, Ciprofloxacin (24% of all patients (8/34), 40% of the patients receiving antibiotics (8/20)) and Moxifloxacin (18% (6/34), 30% (6/20)) were frequently used. In 2006 and 2007 Moxifloxacin was frequently given. 45% (5/11) of the patients with antibiotic treatment in that period received Moxifloxacin. Since suspicions arose that Moxifloxacin triggers CDAD, in an antibiotic stewardship Moxifloxacin wasn't given in routine since 2008. Only one more patient since 2007 developed a CD infection after receiving Moxifloxacin.

Beside antibiotics, PPI, cytostatic agents and tube feeding were common in radiooncological patients.

Of the 18 patients with nutrition via gastric tube, 14 had tube feeding as the only food intake. There was no patient on parenteral nutrition as the only food intake (Table 2).

**Table 2:** Risk Factors

	Age [years]	KPS	Antibiotic treatment	PPI	Cytostatic agents	Tube feeding	Parenteral nutrition	Immuno-suppression
1	85	40%	+	+	-	+	-	-
2	51	60%	-	+	+	+	-	-
3	69	60%	+	+	-	-	-	-
4	71	60%	+	+	-	+	-	-
5	46	90%	-	-	+	-	-	-
6	53	70%	-	+	+	+	+	-
7	67	80%	+	+	+	-	-	-
8	70	70%	-	+	+	+	-	-
9	66	70%	+	+	-	-	-	-
10	63	60%	+	-	+	+	+	-
11	81	60%	+	-	-	-	+	-
12	82	70%	+	+	+	+	-	-
13	70	50%	+	+	-	-	-	-
14	46	80%	-	+	+	-	+	-
15	67	60%	+	+	+	-	-	+
16	60	80%	-	-	+	-	-	-
17	59	60%	+	+	+	-	-	-
18	64	60%	+	+	+	-	-	+
19	68	70%	+	+	+	-	-	+
20	52	70%	-	+	+	+	-	-
21	81	50%	-	+	-	-	-	-
22	56	80%	-	-	+	+	-	-
23	61	70%	-	+	-	+	-	-
24	71	90%	-	-	+	+	-	-
25	74	100%	-	-	+	-	-	-
26	64	50%	-	+	-	+	-	-
27	69	70%	+	+	+	+	-	+
28	70	70%	+	+	-	+	-	-
29	57	90%	+	+	+	-	-	-
30	60	50%	+	+	+	-	-	-
31	85	80%	-	+	+	+	-	-
32	73	60%	+	-	-	+	-	-
33	75	50%	+	+	-	+	-	+
34	63	70%	+	+	+	+	-	-

### Treatment of CDAD

For all patients with CDAD therapy with PPI, antibiotics and cytostatic agents was paused or stopped if possible. In two patients no further antibiotic treatment for CD was necessary. Since 2007 all patients were treated with probiotics (Saccharomyces boulardii) until there were no more clinical symptoms for two days.

Following the guidelines and recommendations of the Robert Koch Institute patients were treated with metronidazole as the first line therapy to avoid vancomycin resistant enterococci. According to the dysphagia of the patients metronidazole was given oral, via gastric tube or parenteral.

Patients with a severe diarrhea, with a KPS of less than 60% or with more than two episodes of CDAD were treated with oral vancomycin as first line therapy.

If the symptoms did not disappear within five days of treatment with metronidazole the therapy was changed to vancomycin. 4 Patients were treated with combination of parenteral metronidazole and enteral vancomycin.

The average treatment time with antibiotics was 15 days (range from 5 to 83 days).

### Arrangements for prophylaxis

Since 2008 the arrangements for prophylaxis have been intensified. Antibiotics and PPI were restricted in use. Tube feeding and parenteral nutrition as the only food intake was avoided. All patients with diarrhea continuing longer than 24 hours and clinical suspicion of having CDAD were isolated prophylactically.

Isolation implicates a single room with a separate bathroom. Gloves and special gowns were used by the staff dealing with the patients on the ward and the staff dealing with the patient at the linear accelerator. Additional hand hygiene with water and soap for all persons exiting the isolation room was performed as well as for the staff dealing with the patient at the linear accelerator. Surface disinfection was intensified with sodium-hypochloride.

In consultation with the Department of medical Microbiology and Hygiene in an antibiotic stewardship Moxifloxacin was not used in clinical routine since 2008.

Patients with four or more risk factors received probiotic medication (Saccharomyces boulardii) during the radiooncological treatment. Those patients with one episode of CDAD received probiotic medication for the remaining radiooncological treatment.

The chronological incidence of CDAD showed one patient of 484 in 2006 (incidence 0,2%), 16 of 398 in 2007 (4,0%), 11 of 423 in 2008 (2,6%), 4 of 384 in 2009 (1,0%) and 2 of 461 in 2010 (0,4%).

### Complications of CDAD

The effect of CDAD on patient treatment showed 24 of 34 patients (73%) with an electrolyte imbalance. Of those patients 15/24 (63%) had an imbalance on two or more electrolytes. 4/34 patients needed intravenous substitution of the electrolytes, the others an oral or enteral substitution.

27/34 patients (79%) developed a hypoproteinemia with a need to treat it in 12/27 (43%) of these patients.

Initially the median KPS of the patients was 70%. After healing of the CDAD the median KPS was 50%.

12/34 patients (35%) had a prolonged hospitalization of 7 days in average (range from two to 75 days). The patient with prolonged inhabitation of 75 days had to be treated on intensive care unit for most of the time.

12/34 patients had more than one episode of CDAD during radiooncological treatment. All of these patients had two episodes except one who had three.

In 14/34 patients (42%) radiotherapy had to be interrupted because of CDAD. This interruption was four days in average (range one to 27 days).

Of those 14 patients with interruption of radiation the average interruption was 10 days. In two patients radiotherapy could not be restarted.

In 21 of the 34 patients a concomitant chemoradiation was planned. For those 21 patients 89 cycles were planned altogether. 43% of the chemotherapy cycle (38 of 89 cycles) could be applicated.

In 62% of the cases (13/21 patients) chemotherapy had to be stopped and could not be continued. Only 19% of the patients (4/21 patients) could receive all of the planned cycles and only 10% of the patients (2/21 patients) could receive all of the planned cycles in time.

In four patients an initially curative concept had to be changed into a palliative treatment concept. A reduced general condition after the CDAD did not allow treating them in the initially planned concept.

Four patients (12%) died due to CDAD. One patient died in a septicaemia. Three patients did not recover adequate after the infection and died of complications. Two of the patients were treated with the combination of metronidazole and vancomycin. One patient was treated with vancomycin and for the last patient treatment was started with enteral metronidazole. This patient refused further therapy of CDAD because of the advanced tumour stage and died of septicaemia.

## Discussion

The incidence in our collective is with 1,6% high compared to data in literature. Reichardt et al. describes an incidence of 0,1% for Europe and an incidence of 0,1% to 2% for the USA [3]. Heinlen et al. could found an incidence of 0,6% for the USA in the year 2003 [28].

Especially the patients with head and neck cancer have a high risk of developing CDAD. This might be due to a multitude of risk factors. 19/34 of our patients were suffering from a squamous cell carcinoma of the head and neck (SCCHN).

Chemoradiation is a standard treatment for different carcinomas [17,29-31]. Many patients on radiooncological wards have at least this as a major risk factor [16,17,32]. Some of these patients develop Neutropenia and are therefore immunocompromised [18,19].

Several underlying diseases, mainly a reduced general condition and compromised immune function have been described as risk factors to develop CDAD [1,11].

Hospitalized radiooncological patients often need an antibiotic or antimycotic medication during radiotherapy [21,22]. Beside those risk factors, the frequency of a treatment with proton pump inhibitor is estimated to be quiet high on radiooncological wards. Tube feeding and parenteral nutrition favour the onset of CDAD [3,33,34].

Especially head and neck cancer patients have a high rate of dysphagia [18]. Nearly all patients having a dysphagia of CTC grade II or higher requiring tube feeding or parenteral nutrition [20,35]. Wolff et al. found a rate of dysphagia in head and neck tumour patients of 77% and a rate of Grad 3 leukopenia or higher of 11% [18]. In our collective all head and neck cancer patients but 3 were addicted to tube feeding or parenteral nutrition (18/21).

There are data identifying underlying diseases of the gastrointestinal tract to be risk factors for CDAD [2,3]. Whether abdominal or pelvine radiotherapy triggers CDAD is not known. In our collective only one patient received abdominal and five patients received pelvic radiotherapy.

Some data give evidence that haematological and oncological patients are patients at risk for developing CDAD [12-14]. Most data exist for paediatric oncological units and for bone marrow transplant units [12,15,36,37]. In the reports on haematology-oncology patients, that include patients undergoing radiotherapy, no subgroup analysis was done for that collective of patients [13]. In summary there are no data about the risk for radiooncological patients suffering from CDAD.

Our data confirm the assumption that radiooncological patients are also patients at risk for an infection with clostridium difficile.

Several authors have shown a decrease in local control for different tumours due to unplanned interruptions of radiotherapy [38,39]. Bese et al. reported a decrease of 1.4% per day of unplanned interruption for head and neck carcinomas. SCCHN are common in our collective and often have an interruption of radiotherapy.

42% of the patients developing a CDAD need a pause of the radiation. For those patients who have an interruption of the radiotherapy, the average interruption time is ten days. A decrease of loco regional control of 10 to 12% for one week of interruption has been reported [38,40].

Beside interruption of radiotherapy patients suffering from CDAD have several additional factors decreasing the feasibility of the oncological treatment like weight loss, incomplete chemotherapy and a decrease of general condition.

For bronchial carcinoma, studies have emphasized the importance of prolonged overall treatment time. One study suspected an increase of the risk of death of 2% even for a one day break [41]. A statistically significant decrease in overall survival for patients receiving a radiochemotherapy for SCLC could be found [38].

Beside bronchial carcinoma and head and neck cancer, also for cervical carcinoma, anal cancer and several other carcinomas it has been reported that prolongation of overall treatment time and interruption of radiotherapy decreases the loco regional control [38]. For that reason, a CDAD is a serious problem for patients undergoing radiotherapy.

Also the feasibility of chemotherapy concomitant to radiotherapy is important for loco regional control and overall survival [42]. Several authors suspect the cumulative dose of concomitant chemotherapy to be an important prognostic factor [43,44]. If you compare the feasibility of chemotherapy in our collective with data in literature, you can find a low cumulative dose for the patients with CDAD [19].

In our collective only 19% of the patients received the complete chemotherapy dose. For example of the patients receiving Cisplatin only 3 of 13 patients received at least 200 mg/m^2 ^Cisplatin. The poor feasibility of chemotherapy in our collective might decrease the loco regional control and overall survival for patients suffering from a CDAD.

In summary with four patients dying due to CDAD, a change from a curative to a palliative concept in another four patients, CDAD is a severe problem for radiooncological patients. Compared with the lethality of 0,5% to 2% described in literature the 11,8% in our collective (four out of 34 patients) seems to be very high [3]. This might be due to a negative selection of the patients. The average age is high and patients had a high number of risk factors, concomitant diseases and often a reduced general condition.

There are several options reducing the risk of developing CDAD. Beside the restrictive use of antibiotics and proton pump inhibitors, the upkeep of oral nutrition as long as possible is essential in reducing the rate of CDAD. Especially Moxifloxacin, which is known to trigger CDAD, was not used since 2008 in clinical routine [7,45]. The high rate of patients in our collective receiving Moxifloxacin until 2007 and the decreasing incidence of CDAD since 2008 seemed to be an effect of discontinuing Moxifloxacin in clinical use.

With these arrangements and an intensive screening for Clostridium difficile the rate of infections has declined from 16 cases in 2007 to 11 in 2008, 4 in 2009 and 2 in 2010.

Even though there is only little evidence in probiotics reducing the rate of CDAD for patients at risk, we treated all patients with four or more risk factors frequently [28,46-49].

## Conclusion

The effect of CDAD on the feasibility of the radiotherapy and a concomitant chemotherapy is remarkable. The morbidity of patients is severe with a high lethality.

Reducing of risk factors, an intense screening and the use of probiotics as prophylaxis can reduce the incidence of CDAD.

## List of abbreviations

CD: Clostridium difficile; CDAD: Clostridium difficile:associated diarrhea; KPS: Karnofsky Performance Status; PPI: Proton pump inhibitor; SCCHN: Squamous cell carcinoma of the head and neck

## Competing interests

The authors declare that they have no competing interests.

## Authors' contributions

MGH planned the design of the study, did identify the patients, register the patients' data, correlate the data and drafted the manuscript. MH was participating in identification of patients data. OK participated in planning of the study and its coordination. All authors read and approved the final manuscript.
